# SARS-CoV-2 Infection and Guillain-Barré Syndrome

**DOI:** 10.3390/pathogens10080936

**Published:** 2021-07-24

**Authors:** Huda Makhluf, Henry Madany

**Affiliations:** 1 Department of Mathematics and Natural Sciences, National University, La Jolla, CA 92037, USA; 2 Public Health, University of California, Irvine, CA 92697, USA; hmadany@uci.edu

**Keywords:** SARS-CoV-2, COVID-19, Guillain-Barré Syndrome

## Abstract

Severe acute respiratory syndrome coronavirus strain 2 (SARS-CoV-2) is a beta-coronavirus that emerged as a global threat and caused a pandemic following its first outbreak in Wuhan, China, in late 2019. SARS-CoV-2 causes COVID-19, a disease ranging from relatively mild to severe illness. Older people and those with many serious underlying medical conditions such as diabetes, heart or lung conditions are at higher risk for developing severe complications from COVID-19 illness. SARS-CoV-2 infections of adults can lead to neurological complications ranging from headaches, loss of taste and smell, to Guillain–Barré syndrome, an autoimmune disease characterized by neurological deficits. Herein we attempt to describe the neurological manifestations of SARS-CoV2 infection with a special focus on Guillain-Barré syndrome.

## 1. Introduction

SARS-CoV-2 is an enveloped, positive-stranded RNA virus belonging to the Coronaviridae family and Betacoronavirus genus. SARS-CoV-2 and the related SARS-CoV-1 and MERS-CoV share several characteristics including severe disease outcomes. SARS-CoV-2 has a ~30 Kb genome encoding 29 proteins, with four structural proteins (envelope, membrane, nucleocapsid, and spike), 16 nonstructural proteins, and nine accessory proteins. However, unlike SARS-CoV-1 and MERS-CoV, which have mortality rates of 10% and 30%, respectively, SARS-CoV-2 appears to have a lower mortality rate of about 0.7%. SARS-CoV-2 spike glycoprotein mediates attachment to host cells via the ACE 2 receptors and is the most exposed and immunogenic [1].

The rapid worldwide spread of SARS-CoV-2 prompted the World Health Organization (WHO) to declare the outbreak of SARS-CoV-2 a global pandemic on 11 March 2020 [2]. According to the most recent report from the WHO (mid-June 2021), the number of COVID-19 cases, the illness resulting from SARS-CoV-2 infections, has globally reached ~177,109,000, including 3,840,223 deaths. Notably, there have been a total of 2,378,482,776 administered COVID-19 vaccine doses worldwide. As of June 2021, SARS-CoV-2 represents the most important human infection in the U.S. with 601,125 deaths reported [3].

SARS-CoV-2 infections typically present with a range of clinical manifestations ranging from fever and chills, cough, shortness of breath, loss of taste and smell, and fatigue, to persistent chest pain and pressure, trouble breathing culminating in acute respiratory distress syndrome and death [4]. There is increased and widespread attention on the neurological complications associated with SARS-CoV-2 infections, which include infections such as headache, dizziness, altered mental state, seizures, ataxia, smell and taste impairments [5,6] and Guillain–Barré syndrome (GBS) [7,8]. Here, we describe various mechanisms in which SARS-CoV-2 could lead to neurologic illnesses, starting with the secondary effects of viral infection and para-infectious illness leading to Guillain-Barré syndrome.

COVID-19 can cause strokes and seizures. Most of the seizures observed in COVID-19 patients have been ischemic in nature, with COVID-19-related hemorrhagic strokes being less common. Possible mechanisms of ischemic cases seen in COVID-19 patients reflect hypercoagulability, endothelial injury, vasculitis, cardiac alterations, systematic hypoxia, thrombosis, and cytokine storm [9]. COVID-19-related neurological symptoms appear to result from an excessive immune response, including cytokine storm, causing bystander damage to the nervous system. It could also be due to direct viral invasion of the nervous system. There are reports of SARS-CoV-2 detection in the CSF and in postmortem brain tissue, the latter even in the absence of respiratory illness [10]. Evidence of direct and widespread neural infection is still being investigated intensely. Loss of smell and taste or anosmia and dysgeusia, respectively, are established neurological manifestations of COVID-19. Initial concerns were raised that the olfactory infection with SARS-CoV-2 may lead to encephalitis and neuroinvasion. Brann et al. demonstrated by bulk and single cell RNA seq that ACE 2 expression was located in olfactory epithelial sustentacular cells and olfactory bulb pericytes and was not detected in the olfactory sensory and bulb neurons [11].

Detection of virus, viral nucleic acid or pentameric IgM specific antibodies from CSF provide direct evidence of neurotropism and neuroinvasion in many viral infections. Evidence, however, substantiating direct invasion of SARS-CoV-2 or infection of the nervous system (neurotropism) is lacking [8]. Severe inflammation and cytokine production can impair brain function. Mehta et al. described a subset of patients with severe COVID-19 suffering from cytokine storm syndromes. A cytokine storm is characterized by increased IL-2, Il-7, GCSF, IFN-γ, TNF-α, and elevated IL-6 levels. Therapeutic options to control the hyperinflammation have included immunosuppressive therapy using steroids, selective cytokine blockade such as tocilizumab and JAK inhibition to avoid fulminant and fatal hypercytokinemia, which typically leads to multiorgan failure and mortality [12].

On the other hand, scientists continue to investigate the effects of autoantibodies in the pathogenesis of SARS-CoV-2. Using Rapid Extracellular Antigen Profiling, a high throughput autoantibody discovery technique, Wang et al. showed that COVID-19 patients exhibited increases in autoantibodies reactivities against cytokines, chemokines, and complement components, perturbing proper immune function and viral control [13]. Autoantibodies against type I IFNs have been identified in 5.2% of hospitalized COVID-19 patients. It remains unknown, however, whether or not these autoantibodies persist beyond the acute phase of the COVID19.

## 2. SARS-CoV-2-Associated GBS

### 2.1. Clinical Characteristics

Guillain-Barré Syndrome (GBS) is an immune mediated neuropathy that affects the peripheral nervous system. Tingling and weakness of the limbs are usually the first signs of GBS followed eventually by paralysis due to the damage caused to peripheral nerve myelin and axons. Cross reactive antibodies from an antecedent infectious antigen are thought to be the culprit. Curated immune epitopes from the Immune Epitope Database from 50 references between 1988 and 2020 encompassed 74 different epitopes from 15 antigenic sources (Supplemental table) including myelin basic protein and ganglioside GM1/GD1a [14].

GBS is difficult to diagnose, especially in its early phases, as signs and symptoms vary from person to person [15]. The precise cause of GBS is still unknown; however, patients often report an associated antecedent infection. The mortality rate is 4–7%. Most recover, with about 60 to 80% of GBS patients walking after six months, with lingering fatigue, weakness, and numbness. GBS may occur in three forms: (1) an acute inflammatory demyelinating polyradiculoneuropathy (AIDP); (2) Miller–Fisher Syndrome (MFS); and (3) acute motor axonal neuropathy (AMAN) and acute motor-sensory axonal neuropathy (AMSAN). AIDP is the most common form in Europe and North America, whereas AMAN and AMSAN are more frequent in Japan, China, and Mexico. Given that GBS affects the myelin sheath integrity of neurons, patients may experience severe complications ranging from difficulties in breathing, if the paralysis spreads to the muscles controlling breathing and respiration, to cardiac arrhythmias and blood pressure fluctuations. Case reports of SARS-CoV-2-related GBS are mounting [16].

The local experiences of SARS-CoV-2 associated-GBS, including its incidence, severity, and mortality rate reported, appear to be different in various geographical areas. Filosto et al. showed a significant increase in GBS cases during the COVID-19 outbreak in Northern Italy [16]. A retrospective study that included data on GBS cases from 12 referral hospitals in the Veneto and Lombardy regions indicated a 2.6-fold increase from an estimated baseline rate of 0.93/100,000/year to 2.43/100,000/year [14]. Additionally, they reported that COVID-19-associated GBS was predominantly demyelinating and was more severe than non-COVID-19 GBS [16]. In Spain, Fragiel et al. reported on 11 GBS cases among 71,904 patients at 61 different Spanish emergency departments, indicating once again a higher relative frequency of GBS 0.15% in COVID-19 patients compared to 0.02% in non-COVID-19 patients [17]. Moreover, they reported that despite higher ICU admissions of “COVID–GBS” patients, the ICU mortality rate was not increased compared to control groups [17]. Zhao et al. reported a case of GBS in Wuhan, China, in which he speculated that the onset of GBS overlapped with the period of SARS-CoV-2 infection, unlike the post-infectious profile typically observed in GBS [18]. Interestingly, this para-infectious onset of GBS parallels the onset observed in Zika-virus-related GBS as well [19]. Toscano et al. reported that the timing between SARS-CoV-2 infection and neurological onset ranged from 5 to 10 days, also suggesting a para-infectious onset [20]. With an increasing number of case reports in the literature and in an effort to investigate the strength and clinical features of the association between GBS and COVID-19, Caress et al. conducted a thorough review of 37 cases of GBS associated with COVID-19. In this retrospective review, 37 patients were analyzed, 65% of which were males and 90% were 50 years or older [21]. More than a third required mechanical ventilation. Time to the nadir of neurologic symptoms in 16 patients with available data ranged from 1.5 to 10 days with a mean time of 5 days. The lack of uniform data collection and reporting and without the use of a precise set of criteria, definitive conclusions are not possible [21]. Additionally, Caress et al. stated that future studies should compare patients with non-COVID-19-GBS with patients with COVID-19-associated GBS in order to establish the incidence of GBS in COVID-19 patients [21].

The distinctive feature of para-infections of COVID-19-associated-GBS as contrasted to the classical postinfection GBS is intriguing. It may be very likely that the elevated levels of IL-6, IL-17, IL-1β, IFN-γ, and TNF-α play an important role in the concurrent and swift progression of GBS [22]. Future research is needed to decipher the possible role of a cytokine storm in COVID-19-associated-GBS and whether corticosteroid treatment would be a prudent approach to manage GBS. Traditionally, corticosteroids are not part of the usual treatment of GBS; however, they may have a role in COVID-19.

### 2.2. In Silico Experiments to Identify the Underlying Mechanisms of GBS

The Zika virus (ZIKV) is a mosquito-borne flavivirus that emerged as a global threat following an outbreak in Brazil in 2014. ZIKV infections of pregnant women are associated with fetal abnormalities such as microcephaly, but of interest here is the fact that infected adults have been known to develop Guillain–Barré syndrome. In 2016, Waldron et al. analyzed regions of homology between the Zika strain MR-766 polyprotein and the human myelin basic protein known to be affected in GBS in an effort to delineate cross-reactive epitopes causing mimicry and a surge in autoimmune reactive cells. Epitopes of Zika virus proteins cross-reactive with human myelin basic protein were found [23]. It remains to be seen, however, whether molecular mimicry might be of great relevance or a mere distractor from the possibility of preexisting autoantibodies that could cause GBS in the context of an exuberant immune response to Zika, SARS-CoV-2, or other infectious agents. In other words, GBS clinical features may be “agnostic” to the type of infection, but could very well be an effect of an immune system in overdrive, where too much of a good thing is bad.

Lucchese et al. deployed the same powerful strategy to identify cross reactive epitopes between human proteins and SARS-CoV-2. They too mined the IEDB to identify curated epitopes within the discovered sequences for validation [24,25]. Using SARS-CoV-2 polyprotein MN908947 and primary amino acid sequences of 41 human sequences associated with neuropathies, they were able to discover two immunologically relevant hexapeptides, KDKKKK and EIPKEE in Heat shock proteins 90 and 60, respectively. KDKKKK mapped to the nucleocapsid protein in SARS-CoV-2 and was present in five experimentally validated epitopes in the IEDB. EIPKEE mapped to Orf1ab and was identified in one experimentally validated epitope in the IEDB—VVTEIPEEKDPGM (Table 1) [24]. These in silico findings warrant further investigation to test the precise molecular mechanisms underlying the immune-mediated neurological damage in SARS-CoV-2-associated GBS.

Additionally, Lucchese et al. investigated the molecular mimicry between SARS-CoV-2 and the respiratory pacemaker neurons in the brain stem known as the pre-Botzinger complex [25]. This complex is essential for the generation of the respiratory rhythm in humans. They hypothesized that damage to this complex by cross reactive epitopes might contribute to the respiratory failure in COVID-19 patients. Mimicry between SARS-CoV-2 proteins and the neuronal proteins dataset of the pre-Botzinger complex was investigated [25]. Remarkably, they discovered three hexapeptides GSQASS, LNEVAK, and SAAEAS in DAB1, AIFM1, and SURF1, respectively (Table 1). AIFM1 and SURF1 (Apoptosis-inducing factor 1 and Surfeit locus protein 1) are involved in neurometabolism. GSQASS and SAAEAS mapped to the nucleocapsid protein in SARS-CoV-2, whereas LNEVAK mapped to the spike protein. Eight curated SARS-CoV-2 epitopes in the IEDB were found to contain these shared amino acid sequences (Table 1). Susceptibility to autoimmune diseases may be influenced by the MHC haplotype of the individual patient as certain MHC class I or II molecules may be more effective than others in peptide presentation and T cell activation. While the in silico approach of investigating the underlying molecular mechanisms of autoimmunity holds great promise, the precise immunological significance and relevance of the identified cross-reactive epitopes will require further study and validation in cell culture and animal models.

## 3. Conclusions

More than a year and a half into the COVID-19 pandemic, it now appears that COVID-19-associated Guillain-Barre is not as common as may initially have been thought. Its clinical and electrodiagnostic patterns moreover seem to mirror pre-pandemic cases. The number of COVID-19 cases in the U.S. has declined sharply, with only 10,399 new cases reported to date [3], largely through effective vaccination campaigns; there remains, however, a dire need for surveillance and tracking of SARS-CoV-2 variants in order to prevent further outbreaks stemming from escape mutants. At present, all vaccine candidates in use have proven effective against SARS-CoV-2 variants (Supplementary Table S1) [26]. Detailed studies are urgently needed to precisely assess the burden of neurologic illness associated and GBS with any new SARS-CoV-2 variant or future strain(s) [26].

GBS had emerged as a significant neurological complication in the earlier large outbreaks of Zika in 2015 and 2016, but not apparently in the ongoing SARS-CoV-2 pandemic. According to a preprint by Singh et al., encephalopathy and cerebrovascular events appear to be the larger group of neurological disease syndromes [27]. The incidence and evolution of GBS in future stages of the pandemic, however, specifically with SARS-CoV-2 variants, remains to be seen, and the advancement of research into and management of GBS must thus remain a priority [28]. The neurological complications associated with SARS-CoV-2 will leave many patients with severe neurological sequelae leading to both personal and societal health, social, and economic burdens [29]. This pandemic has shown us that fruitful collaborations with basic, clinical, and industry partners that employ innovative strategies in artificial intelligence, bioinformatics, machine learning, and agile methodologies will prepare us for the next pandemic. Future studies could provide novel insights into the molecular mimicry mechanisms to support or invalidate the presence of cross-reactive epitopes in COVID-19 patients. Understanding the mechanisms underlying SARS-CoV-2-associated GBS could help in setting the foundation for novel therapeutics and treatment.

## Figures and Tables

**Table 1:** In silico cross reactive SARS-CoV-2 Epitopes.

Hexapeptide	SARS-CoV-2 Protein	Human Protein	IEDB- ID- Epitope
KDKKKK	Nucleocapsid	Heat shock protein 90-beta	30,186- **KDKKKK**TDEAQPLPQRQKKQ 13,680- EPK**KDKKKK**TDEAQPL 33,669- KTFPPTEPKKDKKKK 63,494- TEPK**KDKKKK**TDEAQPLPQRQKK 74,517- YKTFPPTEPK**KDKKKK**
EIPKEE	Orf1ab	60 kDa heat shock protein	112,717- VVT**EIPKEE**KDPGM
GSQASS	Nucleocapsid	Disabled homolog 1	48,067- PKGFYAEGSRG**GSQASS**R 60,669- SRG**GSQASS**RSSSRSR
LNEVAK	Spike	Apoptosis-inducing factor 1	58,640- R**LNEVAK**NL 558,417- EIDR**LNEVAK**NLNESLIDLQELGKYEQY
SAAEAS	Nucleocapsid	Surfeit locus protein 1	31,692- KK**SAAEAS**KKPRQKRTA 31,693- KK**SAAEAS**KKPRQKRTATKQYNVTQ 52,117- QQQGQTVTKK**SAAEAS**KK

## Supplementary Materials

The following are available online at https://www.mdpi.com/article/10.3390/pathogens10080936/s1, Table S1 SARS-CoV2 Variants Supp- GSAID.
